# Anisakid parasite diversity in a pygmy sperm whale, *Kogia breviceps* (Cetacea: Kogiidae) stranded at the edge of its distribution range in the Northeast Atlantic Ocean[Fn FN1]

**DOI:** 10.1051/parasite/2024042

**Published:** 2024-07-30

**Authors:** Paolo Cipriani, Marialetizia Palomba, Lucilla Giulietti, Renato Aco-Alburqueque, Roberta Andolfi, Mariel ten Doeschate, Andrew Brownlow, Nicholas J. Davison, Simonetta Mattiucci

**Affiliations:** 1 Department of Public Health and Infectious Diseases, Section of Parasitology, Sapienza University of Rome Rome Italy; 2 Institute of Marine Research (IMR) Bergen Norway; 3 Department of Ecological and Biological Sciences (DEB), Tuscia University Viterbo Italy; 4 Scottish Marine Animal Scheme, School of Biodiversity, One Health and Veterinary Medicine College of Medical, Veterinary and Life Sciences University of Glasgow Glasgow UK

**Keywords:** *Kogia breviceps*, Pygmy sperm whale, *Anisakis simplex* (s.s.), *Skrjabinisakis paggiae*, *Skrjabinisakis brevispiculata*, *Pseudoterranova ceticola*

## Abstract

Anisakid nematodes are a globally distributed group of marine mammal parasites. Kogiid whales, including the pygmy sperm whale *Kogia breviceps*, host an assemblage of specific anisakid species. Currently, three species are known to be specific to kogiid hosts, i.e., *Skrjabinisakis paggiae*, *S*. *brevispiculata*, and the less studied *Pseudoterranova ceticola*. The aim of this study was to investigate the species diversity of anisakid nematodes sampled from a pygmy sperm whale stranded in 2013 at the edge of its distribution range in the Northeast Atlantic, specifically in the North of Scotland. Nematodes were assigned to genus level based on morphology and identified by sequence analysis of the mtDNA *cox*2 gene and the rDNA ITS region. The present finding represents the first observation of syntopic occurrence of adult stages of *S. brevispiculata*, *S. paggiae*, and *P. ceticola* in a pygmy sperm whale in the Northeast Atlantic, and represent the northernmost record of these species in this area. *Skrjabinisakis brevispiculata* was the most abundant species, accounting for 55% of the identified nematodes, predominantly in the adult stage. *Anisakis simplex* (s.s.) was also abundant, with most specimens in the preadult stage, followed by *S. paggiae* and *P. ceticola.* The pygmy sperm whale is rarely documented in Scottish waters, and its occurrence in the area could suggest expansion of its geographic range. The presence of *S. brevispiculata*, *S. paggiae*, and *P. ceticola* in this whale species in this region may indicate a shift in the whole host community involved in the life cycle of these parasites in northern waters. However, it is also plausible that these parasites were acquired while the whale was feeding in more southern regions, before migrating northbound.

## Abstract (fr)

Les nématodes Anisakidae sont un groupe de parasites de mammifères marins réparti dans le monde entier. Les cétacés Kogiidae, y compris le cachalot pygmée *Kogia breviceps*, hébergent un assemblage d’espèces d’Anisakidae spécifiques. Actuellement, trois espèces sont connues pour être spécifiques aux hôtes Kogiidae, à savoir *Skrjabinisakis paggiae*, *S. brevispiculata* et *Pseudoterranova ceticola*, la moins étudiée. Le but de cette étude était d’étudier la diversité des espèces de nématodes Anisakidae échantillonnés sur un cachalot pygmée échoué en 2013 à la limite de son aire de répartition dans l’Atlantique Nord-Est, plus précisément au nord de l’Écosse. Les nématodes ont été attribués au niveau du genre en fonction de la morphologie et identifiés par analyse de séquence du gène cox2 de l’ADNmt et de la région ITS de l’ADNr. La présente découverte représente la première observation de l’apparition syntopique de stades adultes de *S. brevispiculata*, *S. paggiae* et *P. ceticola* chez un cachalot pygmée dans l’océan Atlantique Nord-Est, et représente le signalement le plus septentrional de ces espèces dans cette zone. *Skrjabinisakis brevispiculata* était l’espèce la plus abondante, représentant 55% des nématodes identifiés, principalement au stade adulte. *Anisakis simplex (s.s.)* était également abondant, la plupart des spécimens étant au stade préadulte, suivi par *S. paggiae* et *P. ceticola*. Le cachalot pygmée est rarement documenté dans les eaux écossaises et sa présence dans la région pourrait suggérer une expansion de son aire de répartition géographique. La présence de *S. brevispiculata*, *S. paggiae* et *P. ceticola* chez cette espèce de cachalot dans cette région peut indiquer un changement dans l’ensemble de la communauté hôte impliquée dans le cycle de vie de ces parasites dans les eaux nordiques. Cependant, il est également plausible que ces parasites aient été acquis alors que le cachalot se nourrissait dans des régions plus au sud, avant de migrer vers le nord.

## Introduction

The pygmy sperm whale, *Kogia breviceps* (de Blainville 1838), and the dwarf sperm whale, *Kogia sima* (Owen 1866), are the only two species in the Kogiidae family, within the superfamily Physeteroidea [16, 38, 41]. These relatively small, elusive, and poorly studied whales have a cosmopolitan distribution in tropical and warm-temperate waters. The pygmy sperm whale primarily inhabits offshore waters beyond the edge of the continental shelf, feeding mainly on cephalopods while occasionally consuming fish and crustaceans at or near the bottom of the sea at a depth of 500–1000 m on the deep shelf or slope [21, 37, 38, 43, 45, 51]. They are found continuously in the Eastern Atlantic, from Argentina to eastern Canada, including the Caribbean Sea, the eastern United States, and the Gulf of Mexico. Sightings and strandings have also been reported widely in the Western Atlantic, from South Africa to Scottish waters [5, 21, 22]. This species has rarely been recorded in Scottish waters. However, its increasing occurrence in the area of the Northeast Atlantic, along with other warm water cetaceans since the late 1980s, has been attributed to rising sea temperatures, as a result of global climate change [22, 28, 45].

Anisakid nematodes are a group of marine mammal parasites with a global distribution. They have complex life cycles, with planktonic or semi-planktonic crustaceans serving as first intermediate hosts, and fish and molluscs as intermediate/paratenic hosts. They develop into their adult stage in marine mammals, predominantly cetaceans for species of the genera *Anisakis* and *Skrjabinisakis,* and pinnipeds and cetaceans for those of *Pseudoterranova*. Recently, the resurrection of the genus *Phocanema* Myers, 1959 has been proposed for the species of the *Pseudoterranova decipiens* (s.l.) complex, i.e., *Ph. decipiens* (s.s.), *Ph. krabbei, Ph. bulbosum*, *Ph. azarasi*, and *Ph. cattani*, maturing at the adult stage in pinnipeds. Thus, according to current systematics, the genus *Pseudoterranova* includes only the two species described in kogiid whales [3], i.e., *P. kogiae* (Johnston & Mawson, 1939) Mozgovoi, 1951 and *P. ceticola* (Deardorff & Overstreet, 1981) Gibson & Colin, 1982. Two other Anisakidae species are known to be specific to kogiid hosts: *Skrjabinisakis brevispiculata* (Dollfus, 1966) Safonova, Voronova & Vainutis, 2021, and *S. paggiae* (Mattiucci, Nascetti, Dailey, Webb, Barros, Cianchi & Bullini, 2005) Safonova, Voronova & Vainutis, 2021. These species have been reported in kogiid whales in several tropical and temperate ocean regions, spanning the Atlantic and Indo-Pacific areas. Their range mostly overlaps with the distribution of their kogiid definitive hosts, with a latitudinal range between 50°S and 50–60°N [2, 7, 12, 14, 24, 30, 35, 40, 46, 48, 49].

The aim of this study was to investigate the anisakid species diversity in an individual pygmy sperm whale stranded at the northern boundary of its distribution range in the Northeast Atlantic, specifically in the North of Scotland. Understanding the parasite assemblage in this host and in this area can provide insights into its recent feeding behavior and migration route, and broaden the knowledge of anisakid parasite distribution.

## Materials and methods

### Sample collection and morphological examination

A sub-adult female pygmy sperm whale *Kogia breviceps* was found live stranded on September 6th, 2013, at Banff, Aberdeenshire, North of Scotland (57°40′13.9″N 2°32′20.8″W). The animal died on the beach. At necropsy, the animal weighed 138 kg and measured 207 cm in length. Nematodes were collected from the cardiac section of the whale’s stomach and stored in 70% ethanol. Once at the laboratory of Sapienza University of Rome, Italy, anisakid nematodes were initially subjected to a morphological examination using light microscopy (Olympus BX51 microscope). All anisakids were measured (length, mm), mature adults were separated from pre-adults based on the presence of caudal papillae and spicules in males, or the presence of eggs in females. Sex was determined whenever possible. The heads and tails were then cut and mounted on glass slides with lactophenol for morphological studies, while body fragments were stored in 70% ethanol for further molecular analyses. The nematodes were assigned to genus level and/or the morphospecies, according to diagnostic morphological features (reviewed by Mattiucci et al. 2018 [30]). Studied characters included the cephalic end, ventriculus length and shape, presence or absence of the intestinal cecum, spicule length and shape, ratio between the right and left spicule lengths (R/L), male caudal end, and arrangement of caudal papillae (reviewed by Mattiucci et al. 2018 [30]).

### Genetic identification of anisakid parasites

A subsample including all the morphospecies of nematodes were genetically identified by sequence analysis of the mitochondrial cytochrome *c* oxidase II (mtDNA *cox*2) gene and the entire internal transcribed spacer (ITS) region of the nuclear ribosomal DNA, including the first internal transcribed spacer (ITS-1), the 5.8S, and the second transcribed spacer (ITS-2).

Total DNA was extracted from ~2 mg of tissue from each specimen using a Quick-gDNA Miniprep Kit (ZYMO Research, Irvine, CA, USA), following the manufacturer’s instructions. DNA extraction was repeated in samples showing poor quality and/or low DNA sample concentration.

For *cox*2 gene sequencing, polymerase chain reaction (PCR) amplification was performed using the primers 211F (5′-TTTTCTAGTTATATAGATTGRTTTYAT-3′) and 210R (5′-CACCAACTCTTAAAATTA TC-3′) [36]. PCR was carried out according to the procedures described by Mattiucci et al., 2014 [31]. The ITS region rDNA (ITS-1, 5.8S rDNA, and ITS-2) was sequenced using the primers NC5 (forward; 5′-GTA GGT GAA CCT GCG GAA GGA TCA TT-3′) and NC2 (reverse; 5′-TTA GTT TCT TTT CCT CCG CT-3′) [54]. PCR was carried out according to the procedures reported in Zhu et al. (2000) [55].

Purification and sequencing of PCR products were carried out by Biofab (Rome, Italy). Sequences were assembled using ChromasPro 2.1.5 (Technelysium Pty Ltd., Tewantin, QLD, Australia) and aligned using ClustalX 2.0 [26]. Identity was checked using the Nucleotide Basic Local Alignment Search Tool (BLAST, www.ncbi.nlm.nih.gov/BLAST).

## Results

### Whale condition

A full necropsy was performed to assess the condition of the whale, including a thorough examination of the stomach lining and contents. Gross examination revealed the animal to be in good nutritional condition. All sections of the stomach were unremarkable with no ulcers noted. Nematodes were present solely in the cardiac section of the stomach, accompanied by a few squid beaks; no marine debris was detected in any stomach sections.

### Anisakid infection

Out of the 95 anisakid nematodes examined, 4 specimens showed morphology consistent with *Pseudoterranova*, with the typical intestinal cecum running along the ventriculus, 35 specimens were assigned to *A. simplex* (s.l.) species complex, showing a long and curved ventriculus, and 56 had morphology consistent with *Skrjabinisakis* spp., showing a short ventricle. Within the 56 specimens with *Skrjabinisakis* spp. morphology, 4 showed a ventriculus with a prominent median narrowing, resembling the violin-shape ventriculus characterising *S. paggiae*. Pre-adult (presence of labia, absence of boring tooth) and adult (showing developed reproductive structures, e.g., caudal papillae and spicules in male worms and ovaries in females) specimens were found in each morphospecies.

### Genetic identification of anisakid nematodes

Readable *cox*2 mtDNA and ITS rDNA sequences were obtained for only 16 specimens, likely due to suboptimal preservation of the nematode samples. Partial *cox*2 mtDNA sequence (580 bp) obtained from a specimen with *A. simplex* (s.l.) morphotype showed 99% identity with *Anisakis simplex* (s.s.) deposited in GenBank (accession number: KC810004). One sequence, obtained from a specimen displaying *Skrjabinisakis* morphotype, matched 99% with the species *S. brevispiculata* (DQ116433). A nematode with *Pseudoterranova* morphotype showed >99% similarity with *P. ceticola* (OP380503). The ITS rDNA sequences obtained from nematodes with *A. simplex* (s.l.) morphotype were identical (100%) and matched 99.88% with *A. simplex* (s.s.) (AB277822). Among the seven ITS rDNA sequences obtained from individuals showing *Skrjabinisakis* morphotype, 6 were identical (100%) to a sequence of *S. brevispiculata* (MK325199), while one, displaying a violin-shaped ventriculus, matched 99.75% with *S. paggiae* (GU295973).

*Skrjabinisakis brevispiculata* was the most abundant species, accounting for 55% of the identified nematodes. The specimens were found in different developmental stages, with the majority being female (65%), ranging from fully mature adults with developed eggs (49 mm) to immature pre-adults (25 mm). Two male specimens of *S. brevispiculata* measuring up to 39 mm displayed well-developed spicules.

All four *Pseudoterranova* specimens (representing 4% of the identified nematodes) were female, with a mean length of 10.3 mm. Although they exhibited adult features, ovaries appeared not fully developed. *Skrjabinisakis paggiae* represented the 4% of identified nematodes. All specimens were female; one measured 37 mm in length and showed ovaries with eggs, while the immature specimens had a mean length of 20.6 mm. The relative frequency of *A. simplex* (s.s.) was 37%. All worms assigned to the *A. simplex* (s.l.) morphotype were in a pre-adult developmental stage, with a mean length below 25 mm, except for one male specimen measuring 30 mm, which possessed developed male spicules.

The DNA sequences of the presently identified *Anisakis* species were deposited in GenBank under the following accession numbers: mtDNA *cox*2: PP888191 (*A. simplex* (s.s.)); PP888189 (*S. brevispiculata*); PP888190 (*P. ceticola*). ITS rDNA: PP884102 (*A. simplex* (s.s.)); PP884101 (*S. brevispiculata*); PP884100 (*S. paggiae*).

## Discussion

Kogiid whales are found worldwide in warm-temperate waters and are known hosts for specific anisakid parasites. This study provides data on the anisakid species diversity from a pygmy sperm whale stranded at the northern distribution limit of this species, harbouring syntopically adult specimens of four nematode species, i.e., *A. simplex* (s.s.)*, Skrjabinisakis brevispiculata*, *S. paggiae*, and *Pseudoterranova ceticola*.

*Skrjabinisakis brevispiculata*, *S. paggiae*, and *P. ceticola* were originally described at the adult stage in kogiid whales [10, 19, 34]. Since then, adult specimens of these nematode species have solely been found in these definitive hosts [2, 7, 12, 24, 32, 40, 46, 48, 49], suggesting host-parasite adaptation of these anisakid species to this cetacean group.

Nematode species were recorded with different relative proportions: *S. brevispiculata* was the most common anisakid nematode identified in the pygmy sperm whale in this study, with an assemblage of a few pre-adult stages and mature individuals. Mature males and females showed sizes comparable with those provided with the species description [33], thus indicating full development of the parasite in its host. The current findings represent the northernmost record (57°40′13.9″N) of *S. brevispiculata* in the Northeast Atlantic. So far, this species was found in kogiids from warm and temperate waters of the Atlantic and Pacific Oceans, and off the Spanish coast of the Northeast Atlantic [2, 34]. *Skrjabinisakis brevispiculata* larvae have rarely been found, at low intensity, in fish from the North Atlantic (reviewed in Mattiucci et al., 2018 [30]), and never in northern seas. The species was recently detected in mesopelagic fish species in tropical areas of the Indian Ocean, suggesting that myctophids play a role in the transmission of this species as direct prey for kogiids or as prey for cephalopods, which themselves represent kogiids prey [6].

The finding of *S. paggiae* nematodes in this study represents the first report of the adult stage in the Northeast Atlantic. Adults *S. paggiae* have been reported in pygmy sperm whales stranded in warm tropical waters of the Central Eastern Atlantic Ocean (Caribbean Sea and Florida coast), and in New Caledonia, Southwest Pacific Ocean [7, 14, 33]. At larval stage, *S. paggiae* has been reported from the common fangtooth (*Anoplogaster cornuta*) in the Irminger Sea (Northeast Atlantic Ocean) [23], from the European hake *Merluccius merluccius* in Moroccan and Mauritanian Atlantic waters [29], and in the swordfish *Xiphias gladius* in tropical equatorial waters of the Atlantic Ocean, in syntopic infection with *S. physeteris* and *S. brevispiculata* [13].

The occurrence of *P. ceticola* in a pygmy sperm whale at such a northern latitude (57°N) represents a rare finding. The specimens, all females, were not fully mature worms, with a size far smaller than reported for adult mature females [10, 19]. The early developmental stages of the worms could suggest they were recently acquired by the host. Adult specimens of this species have been reported in a pygmy sperm whale stranded on the Iberian coast of the Northeast Atlantic [1, 2], in tropical waters of the Central Eastern Atlantic Ocean (Caribbean Sea and Florida coast) [7, 53], and in warm Australian waters [49]. At larval stages, this species has been identified only in mesopelagic and bathypelagic fish species from tropical warm/temperate waters (revised in Bao et al, 2022 [4]).

The occurrence of larval stages of *S. paggiae* in mesopelagic fish [23], *S. brevispiculata* in myctophids [6], and *P. ceticola* in mesopelagic and bathypelagic fish species [4] suggests that these species interact with hosts that develop in the deep-water or meso-/bathypelagic realms of marine ecosystems. Further studies on mesopelagic and bathypelagic fauna in the Northeast Atlantic could shed light on the intermediate/transport hosts for these species, thus elucidating the life cycles of these parasites in these northern waters.

*Anisakis simplex* (s.s.) is a common *Anisakis* species in the Northeast Atlantic area. Adult individuals of this species are found in cetacean species, mainly Mysticetes and Delphinidae [9, 18, 25, 31, 39, 42, 52], whereas L3 stage larva commonly infect many fish and cephalopod species, commonly at high latitudes [8, 30, 47]. The predominance of immature specimens of *A. simplex* (s.s.) recorded in this pygmy sperm whale, with only one adult male specimen out of 35, may suggest that this parasite has low fitness in this host. A correlation between the size of this parasite and its fitness has indeed been documented [15, 52], indicating the ability of *A. simplex* (s.s.) to attain bigger sizes, and eggs production, in killer whales and mysticetes, rather than in dolphins. Previous studies suggested that kogiid whales can acquire *Anisakis simplex* (s.l.) by preying on infected fish and squid, but these species rarely develop into adults [9, 44], suggesting that these cetaceans may represent an accidental host for this parasite species complex. One possible explanation for the relatively high infection by *A. simplex* (s.s.) recorded in this pygmy sperm whale, is that the cetacean at the northern limit of its distribution range struggled to find its usual prey species, and opportunistically preyed on pelagic fish and squid species that commonly harbor high burdens of *A. simplex* (s.s.) in the area [20, 37, 43, 45, 51]. In fact, *A. simplex* (s.s.) has been reported at high infection rates in European flying squid (*Todarodes sagittatus*) off Scotland [8] and in blue whiting (*Micromesistius poutassou*) [27, 47]. Both this cephalopod and this pelagic fish species has occasionally been reported as prey of pygmy sperm whales in the Northeast Atlantic [11, 45].

The results obtained demonstrate a strong host-parasite relationship between pygmy sperm whales and several anisakid species (i.e., *S. brevispiculata*, *S. paggiae*, and *P. ceticola*). This relationship, which involves multiple intermediate/paratenic hosts, reflects a long co-evolutionary history, wherein the ecology of both host and parasite species has been shaped by distribution, diet, and mutual interactions, potentially adapting to the meso- and bathypelagic ecosystems. However, their preference for either of the two *Kogia* species, and their life cycles remain partially unknown. The disorders associated with these nematode infections in their definitive hosts are also poorly known. Ulcers, often linked to clusters of *A. simplex* (s.l.) larvae, are frequently observed in extensive infections among delphinids and mysticetes, although occurrences in physeterids are comparatively rare [1, 15, 17, 39, 42, 49, 50]. There were no ulcers present in any section of the stomach of the stranded pigmy sperm whale here studied, despite the presence of several anisakid species.

The pygmy sperm whale is distributed in deep oceanic waters of the tropical and temperate Atlantic, Indian and Pacific Oceans, from approximately 50°S to 50–60°N [21]. This species has rarely been recorded in Scottish waters, and its occurrence in the area has been interpreted as a shift in the whole community composition comprising whales and their preys, possibly associated with climate change [28, 45]. This phenomenon has been observed and reported in several cetacean species in recent decades, showing a poleward shift following their preferred sea surface temperatures and prey availability to higher latitudes, and forecasting a further shift in their distribution to higher latitudes [28, 45, 54].

The observed occurrence of certain specific parasites in this whale species, such as *S. brevispiculata, S. paggiae,* and *P. ceticola,* previously recorded at the adult stage in tropical-temperate waters, and for the first time recorded at these latitudes, may have several explanations. Most likely this animal, before stranding at northern latitudes, was foraging in more southern regions, which are endemic areas for these anisakid species. Both the host and these parasites have been previously reported from Spanish Atlantic waters [2]. The lifespan of an adult anisakid in a definitive host has been estimated to be 1–2 months [52]; therefore, the animal would have had enough time to acquire the worms while feeding in southern waters and carry them until the stranding. Even though the presence of both pre-adult and adult stages of *S. brevispiculata* and *S. paggiae* could suggest that the animal acquired these nematodes at different times through its feeding activities during its last lifespan, some of them were possibly acquired recently before stranding, thus at northern latitudes. A second hypothesis, supporting the recent observations that kogiid whales might be spreading northward, most likely due to climate change [28, 45], would suggest that the entire host community involving kogiid preys and their parasites are also shifting northwards, finding favorable abiotic and biotic conditions. According to Klimpel et al., [23], *S. paggiae* is present in non-migratory mesopelagic fish species at northern latitudes, probably indicating that early larval stages of this parasite species also found abiotic and biotic conditions permitting them to develop and infect intermediate and paratenic hosts. This suggests that the range of pygmy sperm whales could extend much further north than currently expected, up to 62°N [23].

Further studies investigating these elusive species will shed light on their distribution range and the complex life cycles of their specific parasites.
